# Specific detection of Muscovy duck parvovirus infection by TaqMan-based real-time PCR assay

**DOI:** 10.1186/s12917-018-1600-3

**Published:** 2018-09-03

**Authors:** Chunhe Wan, Cuiteng Chen, Longfei Cheng, Hongmei Chen, Qiuling Fu, Shaohua Shi, Guanghua Fu, Rongchang Liu, Yu Huang

**Affiliations:** Fujian Provincial Key Laboratory for Avian Diseases Control and Prevention, Fujian Animal Diseases Control Technology Development Center, Institute of Animal Husbandry and Veterinary Medicine of Fujian Academy of Agricultural Sciences, Xi-feng Road No.100, Jiantian village, Jin’an district, Fuzhou, 350013 China

**Keywords:** MDPV, NS gene, Specific detection, TaqMan-based real-time PCR assay, Vertical transmission

## Abstract

**Background:**

Muscovy duck parvovirus (MDPV) causes high mortality and morbidity in Muscovy ducks, with the pathogenesis of the virus still unknown in many respects. Specific MDPV detection is often rife with false positive results because of high identity at the genomic nucleotide level and antigenic similarity with goose parvovirus (GPV). The objective of this study was to develop a sensitive, highly specific, and repeatable TaqMan-based real-time PCR (qPCR) assay for facilitating the molecular detection of MDPV.

**Results:**

The specific primers and probe were designed based on the conserved regions within MDPVs, but there was a variation in GPVs of the nonstructural (NS) genes after genetic comparison. After the optimization of qPCR conditions, the detection limit of this qPCR assay was 29.7 copies/μl. The assay was highly specific for the detection of MDPV, and no cross-reactivity was observed with other non-targeted duck-derived pathogens. Intra- and inter-assay variability was less than 2.21%, means a high degree of repeatability. The diagnostic applicability of the qPCR assay was proven that MDPV-positive can be found in cloacal swabs samples, Muscovy duck embryos and newly hatched Muscovy ducklings.

**Conclusions:**

Our data provided incidents that MDPV could be possible vertically transmitted from breeder Muscovy ducks to Muscovy ducklings. The developed qPCR assay in the study could be a reliable and specific tool for epidemiological surveillance and pathogenesis studies of MDPV.

## Background

The family *Parvoviridae* is comprised of two subfamily members, *Densovirinae* and *Parvovirinae*. The subfamily *Parvovirinae* contains eight distinct genera: *Amdoparvovirus, Aveparvovirus, Bocaparvovirus, Copiparvovirus, Dependoparvovirus, Erythroparvovirus, Protoparvovirus,* and *Tetraparvovirus*. At present, the International Committee on Taxonomy of Viruses (ICTV) has classified Muscovy duck parvovirus (MDPV) and goose parvovirus (GPV) as a single species (namely, *Anseriform dependoparvovirus* 1), which is classified into the genus *Dependoparvovirus* in subfamily *Parvovirinae* due to similar genetic properties and evolutionary origins (https://talk.ictvonline.org/taxonomy/).

Reportedly, the genomes of MDPVs and GPVs contain a single copy of the linear, single-stranded DNA genome of approximately 5100 nucleotides in length. The genomes of these viruses are flanked by identical inverted terminal repeats (ITR) at both the 5′- and 3′-terminus. The ITR can fold on itself to form a palindromic hairpin structure. A terminal resolving site (TRS), Rep protein binding site (RBS), and transcription factor binding sites can be found in ITR, which were involved in viral replication, packaging and transcription. There are two major open reading frames (ORFs) in both MDPVs and GPVs genome. The left ORF that encodes for the nonstructural (NS) protein, which is involved in viral replication and regulatory function. However, the right ORF that produces three capsid proteins (VP1, VP2 and VP3), which plays important roles in virus tropism, host range, and pathogenicity. In addition, VP2 and VP3 contain the same carboxyl-terminal portion of VP1 of the viruses, which was generated by differential alternative splicing of mRNA [1–8].

Normally, GPVs have been found in goslings, Muscovy ducklings [5, 8], swans [9], and *Anser cygnoides* [10], whereas MDPV has only been discovered in Muscovy ducklings. MDPV infection was initially described by Professor Lin in our laboratory in the early 1980s [11]. Muscovy ducklings infected by MDPV were characterized by watery diarrhea, wheezing, and locomotor dysfunction. MDPV is mainly observed in Muscovy ducklings less than three-week-old, with the mortality rate reaching as high as 80% depending on age [3, 7, 11].Then the disease was widespread in China, leading huge economic loss to waterfowl husbandry due to the high mortality and morbidity.

A previous study revealed that MDPV and GPV are also pathogenic to Muscovy ducklings, even in the same Muscovy duck flocks [8, 12]. Compared with the GPV virulent strain B and MDPV virulent strain FM, they share more than 80.0% nucleotide similarity at the genome level. In addition, these two viruses exhibit nucleotides and amino acids identities of 83.0 and 90.6% at NS gene level, and 81.5% and 87.6% at VP1 gene level, respectively [1–3]. The high identity at the amino acids level of the VP1 protein indicates potential immunogenic cross-reactivity between MDPVs and GPVs [13–15]. Therefore, differentiating between MDPV and GPV in Muscovy ducklings is essential. Nevertheless, the high homologies in nucleotide identities and immunogenic cross-reactivity between MDPVs and GPVs, increases the risk of omissive and mistaken diagnoses for the specific detection of MDPV.

Recently, a TaqMan-based real-time quantitative PCR (qPCR) assays have gained wide acceptance due to their rapid nature, sensitivity, reproducibility, and the reduced risk of carry-over contamination as a result of the specific TaqMan probe, which had been widely used for viral epidemiological surveillance and pathogenesis studies [16–19]. Thereby, the aim of this study was to design a fully validated, reliable, and highly specific TaqMan-based real-time quantitative PCR assay for precise detection of MDPV infection based on specific primers and probe, designed by targeting the conserved region of the MDPV NS gene after bioinformatics analysis.

## Methods

### Viruses and bacteria strains

Avian influenza virus (H9N2 AIV), avian Tembusu virus (ATmV), duck hepatitis virus type 1 and 3 (DHAV-1 and DHAV-3), Muscovy duck reovirus (MDRV), duck adenovirus A (DAdV-A), duck enteritis virus (DEV), Muscovy duck origin goose parvovirus (GPV), novel goose parvovirus (N-GPV), *Escherichia coli* (*E. coli.*), Pasteurellamultocida (*P.M.),* Rimerella anatipstifer (*R.A.*) and Salmonella spp. (*S.S.*) were isolated and kept in our laboratory, which was decribed the same as previously reported [12, 20, 21].

### Nucleic acids extraction and cDNAs preparation

Viral RNAs (RNA viruses, i.e. AIV, ATmV, DHAV-1 and DHAV-3, MDRV) were extracted using EasyPure Viral DNA/RNA Kit (Tiangen, Beijing, China), viral DNAs (DNA viruses, i.e. DAdV-A, DEV, GPV and N-GPV) were extracted using EasyPure Micro Genomic DNA Kit (Transgen Bioteck, Beijing, China), according to instructions provided by the manufacturer. cDNAs (AIV, ATmV, DHAV-1 and DHAV-3, MDRV) were prepared with viral RNAs (with approximate 100 ng of viral RNA) using TransScript II All-in-One First-Strand cDNA Synthesis SuperMix (One-Step gDNA Removal) (Tiangen, Beijing, China). Bacteria genomic DNAs (bacteria pathogens, i.e. *E. coli.*, *P.M.*, *R.A*. and *S.S.*) were extracted using EasyPure Bacteria Genomic DNA Kit (Transgen, Beijing, China). DNAs and cDNAs were then quantified using a NANODROP 2000 spectrophotometer (Thermo Scientific, Waltham, MA, USA). All extracted DNAs and cDNAs templates were stored at − 80 °C until use.

### Primers and probe selection and design

Primer and probe selection was performed on the evolutionarily most conserved regions of the NS gene of MDPV. Briefly, a total of 15 MDPVs and 37 GPVs NS gene sequences were downloaded from the GenBank database (https://www.ncbi.nlm.nih.gov/nucleotide/), and these 52 NS gene sequences were aligned using the Lasergene package MegAlign program by ClustalW method. The identification of the conserved region, which was highly conserved in MDPVs, but there was a characteristics variation in GPVs. The obvious different region between MDPVs with GPVs, was selected for the primers and TaqMan probe design. The forward primer MDPV-qF (5’-TACGAATGAACAAACCAA-3′), the reverse primer MDPV-qR (5′- CGCTCTTAATATCTCCTCTA-3′), and the TaqMan probe MDPV-qP (FAM-5′- TGAACGAGCGAATGAGCCTTCC-3′-Eclipse) were designed using Primer Premier Software version 5.0 (Premier Biosoft, Palo Alto, CA, USA). The length of amplicon was 118 base pairs (bp). Primers (MDPV-qF and MDPV-qR) and probe (MDPV-qP) were verified by Basic Local Alignment Search Tool (BLAST, https://blast.ncbi.nlm.nih.gov/Blast.cgi) for specificity analysis, then these verified primers and probe were synthesized by a commercial company (TaKaRa, Dalian, China).

### Construction of recombinant plasmid containing the NS gene of MDPV

The NS gene (1884 bp) of MDPV (strain FJM5) [8] was amplified by PCR, with the primer sets of forward primer (NSF) 5’-ATGGCATTTTCTAGGCCTCTTCA-3′ and reverse primer (NSR) 5’-TTATTGTTCATTCTCCATATCAT-3′. A conventional PCR was performed in a Thermal Cycler Dice (TaKaRa, OTSU, SHIGA, Japan), the isolated DNAs were used as template in a reaction volume of 50 μl reaction mixture containing 25 μl ThermoScientific DreamTaq Green PCR Master Mix (2×) (Thermo Fisher Scientific Inc., Shanghai, China), 1 μl primers NSF and NSR (20 μM each), 1 μl DNA template, and 22 μl Nuclease-free water. The PCR reaction was conducted using a program that included with an initial denaturation at 94 °C for 5 min, followed by 35 cycles of denaturation at 94 °C for 50 s, annealing at 53 °C for 35 s, elongation at 72 °C for 120 s, and then a final extension at 72 °C for 10 min.

The amplified PCR products were then subjected to electrophoresis on 1.0% agarose gels for analysis. The expected PCR amplicons were purified and then T-A cloned using the pMD18-T Vector Cloning Kit (TaKaRa, Dalian, China). Then the transformants were identified by a commercial company (Sangon, Shanghai, China) for nucleotide sequencing. The selected recombinant plasmid, pMD18-NS, was used as the standard plasmid. The plasmid pMD18-NS was then quantified using a NANODROP 2000 spectrophotometer (Thermo Scientific, Waltham, MA, USA). According to the formula described by Yun et al. [22], the copy numbers of the plasmid pMD18-NS was calculated. Serial 10-fold dilutions of plasmid pMD18-NS, were diluted using EASY Dilution (TaKaRa, Dalian, China). All the serial diluted plasmids (ranging from 2.97 × 10^7^ to 2.97 × 10^0^ copies/μl), were stored at − 80 °C until use.

### Real-time PCR assay

Real-time PCR amplification and detection were carried out on Mastercycler ep realplex (Eppendorf, Germany). The concentration of the primers, probe, and templates were optimized based on the obtained fluorescence and lowest threshold cycle (Ct). The optimized TaqMan-based PCR was prepared in a final volume of 25 μl containing 1 μl DNA template, 12.5 μl Premix Ex Taq (Probe qPCR, TaKaRa, Dalian, China), 0.5 μl of each primer (MDPV-qF and MDPV-qR, 10 μmol/l each), 2 μl probe (MDPV-qP, 5 μmol/l), and 8.5 μl Nuclease-free water to to adjust the reaction volume (total reaction volume of 25 μl). The mixed reactions was conducted in a single tube in a Mastercycler ep realplex by using the following thermoprofile: 40 cycles of 95 °C for 5 s, 58 °C for 10 s, and 72 °C for 15 s. 10-fold serial dilutions of plasmid pMD18-NS, containing different copy numbers of DNA (2.97 × 10^6^ to 2.97 ×  10^1^ copies/μl) were conducted to generate the standard curve. All of the reactions were conducted in triplicate simultaneously. Analysis of each assay was conducted with CalQplex software (Mastercycler ep realplex, Eppendorf, Germany) according to the instruction manual. The software automatically uses the Ct values of serial dilutions of standards to calculate a standard curve, which shows the Ct values as a function of the amount of different copy numbers of DNA.

### Sensitivity analysis

To evaluate the limit of detection (LOD) of the qPCR assay, 10-fold serial dilutions of plasmid DNA standard (ranging from 2.97 ×  10^5^ to 2.97 × 10^0^ copies/μl) were prepared to determine the sensitivity. Each concentration was run in triplicate. Meanwhile, conventional PCR (cPCR) was performed with primers (NSF1 and NSR1) (NSF1, 5′- CAATGGGCTTTTACCAATATGC-3′ and NSR1, 5′- ATTTTTCCCTCCTCCCACCA-3′) and the same standard plasmid, in order to determine the LOD of cPCR assay. The cPCR reaction mixtures and thermal profile was described the same as previously reported [12]. PCR products were visualized following electrophoresis of 5 μl of each reaction in a 1.0% agarose gel according to instructions provided by the manufacturer. The LOD between the cPCR and qPCR assay were then compared.

### Specificity analysis and reproducibility analysis

To determine the specificity of the qPCR assay, ten ng of extracted DNAs and cDNAs templates were used for the specificity analysis. The qPCR assay was carried out in triplicate to amplify a panel of duck-derived pathogens, i.e. H9N2 AIV, ATmV, DHAV-1, DHAV-3, MDRV, DAdV-A, DEV, GPV, N-GPV, *E. coli.*, *P.M.*, *R.A.* and *S.S*.. Nuclease-free water also used in the qPCR run to validate the specificity of qPCR assay as negative control. For reproducibility analysis of the qPCR assay, the 10-fold dilutions of pMD18-NS (concentration with 2.97 × 10^5^, 2.97 ×  10^3^, and 2.97× 10^1^ copies/μl) were tested to evaluate the coefficient of variation (CV). For intra-assay variability of qPCR assay, triplicates of each dilution were analyzed, and the CVs were calculated according to the formula of the geometric mean Ct values deviation. For inter-assay variability, a coefficient of variation which expresses the standardized measure of dispersion with different time.

## Clinical samples detection of MDPV

### Detection of MDPV in field samples

In order to validate the qPCR assay, a total of 75 individuals Muscovy duckling-origin cloacal swabs samples (less than three-week) with diarrheal symptoms were collected in Fujian, Jiangxi, Guangdong, Jiangsu, and Zhejiang provinces, China. All of the suspensions were subjected to three freeze-thaw cycles and then centrifuged at 8000 rpm at 4 °C for 30 min. Viral DNAs were extracted from the harvested supernatants using EasyPure Micro Genomic DNA Kit (Transgen Bioteck, Beijing, China). Conventional PCR was preformed simultaneously.

### Detection of MDPV in Muscovy duck embryos and newly hatched ducklings

Previous study indicates possible vertical transmission of N-GPV and suggests that N-GPV may be transmitted from breeder ducks to ducklings in *ovo* [23]. In order to determine the hypothesis whether MDPV could be possible vertically transmitted or not, 20 Muscovy duck embryos (15-day post fertilization) and 20 newly hatched Muscovy ducklings (1-day-old) were collected from the diseased farms where the virus (FJM5 strain) was discovered [8]. The liver of each embryo and newly hatched duckling was pooled and regarded as one sample. These samples were homogenized in phosphate-buffered saline (PBS) (20%, *w*/*v*). Viral DNAs were extracted from tissue homogenates of liver using EasyPure Micro Genomic DNA Kit (Transgen Bioteck, Beijing, China). Conventional PCR was preformed simultaneously. All of the cPCR-positive amplicons were harvested, T-A cloned, and then sequenced to verify the results for Sanger seuquencing at Sangon (Shanghai, China) in both directions.

## Results

### NS gene analysis

We compared a total of 52 NS gene sequences (including 15 MDPVs and 37 GPVs) downloaded from the GenBank database. We found that within the MDPV cluster, there was a higher nucleotide identity (more or equal than 98.0%) than within the GPV cluster (more or equal than 93.3%). In addition, the NS gene homology between GPV cluster and MDPV cluster ranged from 80.8 to 83.4% **(**Table 1). The primers MDPV-qF and MDPV-qR, and the TaqMan probe MDPV-qP variation within MDPVs are listed in Table 2. Moreover, the 3′-terminal of MDPV-qP at position 1521–1524 was TTCC, while the sequence at this position in the 37 GPVs was either *GGAG* (54.05%, 20/37), *AGAG* (40.54%, 15/37), *AGAT* (2.70%, 1/37), or *GAAG* (2.70%, 1/37). This data demonstrated that sequences at positions 1521–1524 were significantly different between MDPVs and GPVs, which can be chosen for specific probe design.

**Table 1:** Percentage nucleotide (nt) identity within and between MDPVs and GPVs

Viruses	Winthin MDPVs	Winthin GPVs	Between MDPVs and GPVs
Percentage	98.0–100%	93.3%--100%	80.8–83.4%

**Table 2:** Sequence variation in multiple sequences alignment

Primers	Sequence(5′ → 3′)^**b1**^	Frequency	GenBank accession numbers MDPVs^**b2**^
MDPV-qF	TACGAATGAACAAACCAA	80.0% (12/15)	/
	**C**ACGAATGAACAAACCAA	13.33% (2/15)	KU844281, JF926697
	TACGAATGAAC**G**AACCAA	6.67% (1/15)	KC171936
MDPV-qR	CGCTCTTAATATCTCCTCTA	93.33% (14/15)	/
	TAGA**A**GAGATATTAAGAGCG	6.67% (1/15)	KT865605
MDPV-qP	TGAACGAGCGAATGAGCCTTCC	100% (15/15)	/

^b1^The variations are marked as Bold and underline

^b2^The GenBank accession numbers of MDPV strains used in this study are as follows: U22967, KU844282, KX000918, JF926698, KM093740, KY744743, KY069274, JF926695, KY511293, JF926696, X75093, KU844281, JF926697, KC171936, and KT865605. Only variations from GenBank accession numbers of MDPV strains have been marked

### Real-time PCR

**Fig. 1:**
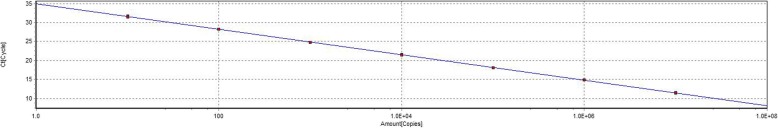
Standard curve of TaqMan-based real-time PCR assay

**Fig. 2:**
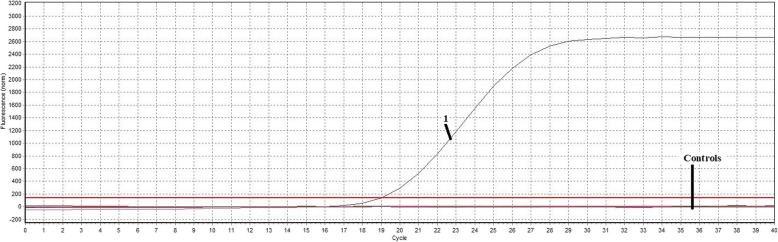
Specificity of TaqMan-based real-time PCR assay. 1: MDPV; Controls: H9N2 AIV, ATmV, DHAV-1, DHAV-3, MDRV, DAdV-A, DEV, GPV, N-GPV, *E. coli.*, *P.M.*, *R.A.*, *S.S*. and Nuclease-free water. These controls were all found with no fluorescence signal

**Fig. 3:**
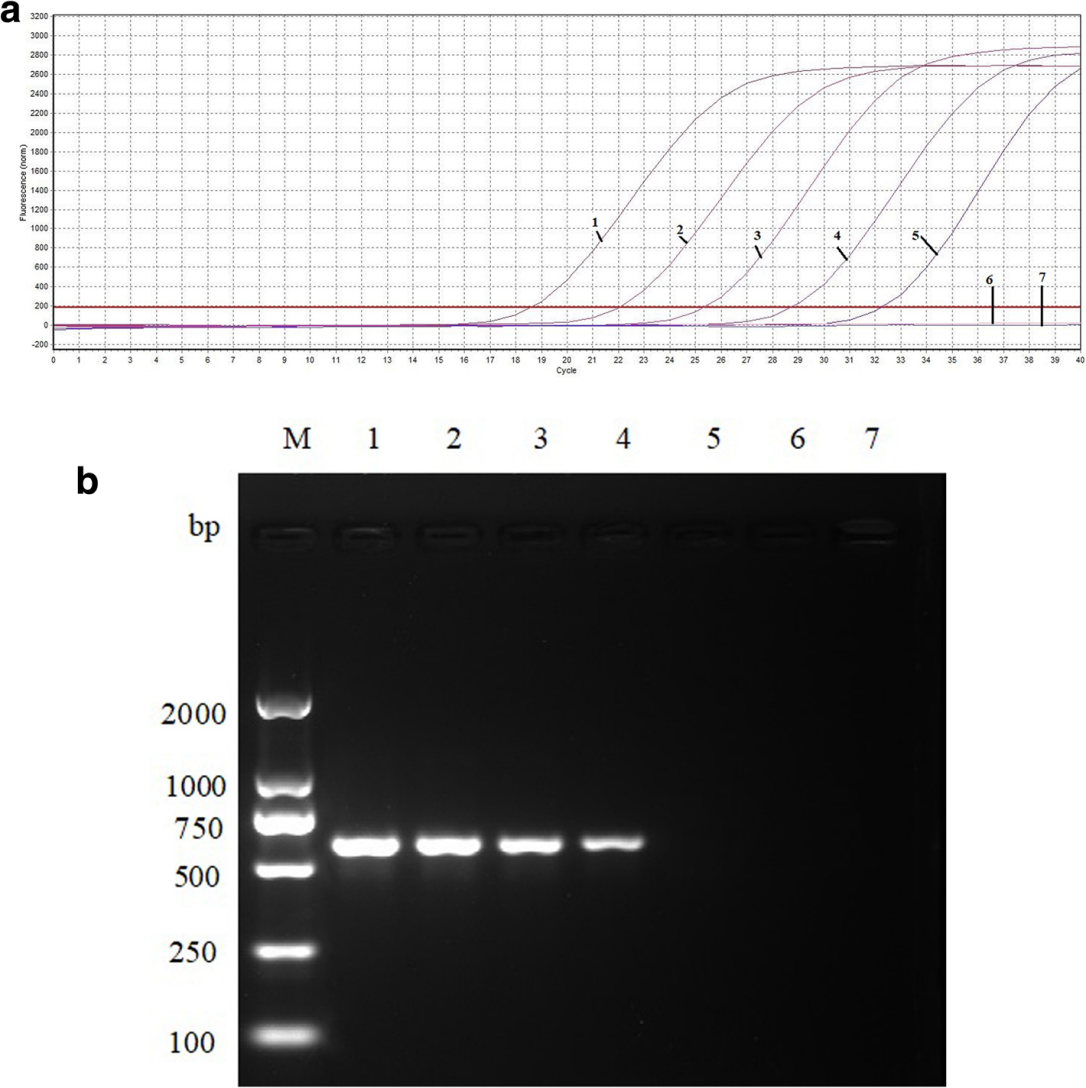
**a** Sensitivity of TaqMan-based real-time PCR assay for MDPV detection. **b** Sensitivity of conventional PCR assay for MDPV detection. 1–6: a serial of ten-fold dilutions plasmid DNA (2.97 × 10^5^ to 2.97 × 10^0^ copies/μl); 7: negative control; M: DL2000 DNA Marker

The generated standard curve showed linearity over the 2.97 × 10^6^ to 2.97 × 10^1^ copies/μl range, with a slope of − 3.358 and the Y-intercept was 34.93, efficiency of 0.99, which was conducted by software of Mastercycler ep realplex (Eppendorf, Germany) (Fig. 1). Using the viral DNAs, cDNAs, bacterial DNAs and Nuclease-free water, the results showed that only MDPV was detected by the TaqMan-based real-time PCR assay. No fluorescence signal was detected in control samples of H9N2 AIV, ATmV, DHAV-1, DHAV-3, MDRV, DAdV-A, DEV, GPV, N-GPV, *E. coli.*, *P.M.*, *R.A.*, *S.S*. and Nuclease-free water (Fig. 2), which indicated the excellent specificity of the qPCR assay. The LOD of the qPCR assay was 29.7 copies/μl (Fig. 3a). In contrast, conventional PCR had a detection limit of 2.97 × 10^2^ copies/μl (Fig. 3b). The intra-assay CVs of pMD18-NS (concentration with 2.97 × 10^5^, 2.97 × 10^3^, and 2.97× 10^1^ copies/μl) were 0.59%, 0.44% and 1.43%. The inter-assay CVs were 0.66%, 0.63% and 2.21% (listed in Table 3), which indicate a high degree of repeatability.

**Table 3:** Intra- and inter-assay reproducibility for TaqMan-based PCR

Concentration of standard plasmid (copies/μl)	Intra-assay variability		Inter-assay variability	
	$\overline{X}\pm SD$	CV (%)	$\overline{X}\pm SD$	CV (%)
2.97 × 10^5^	18.65 ± 0.11	0.59	18.72 ± 0.12	0.66
2.97 × 10^3^	25.40 ± 0.11	0.44	25.46 ± 0.16	0.63
2.97 × 10^1^	32.32 ± 0.46	1.43	32.48 ± 0.72	2.21

### Clinical samples application

Senventy-five cloacal swabs from Muscovy ducklings with diarrhea were evaluated of MDPV diagnosis by the TaqMan-based real-time PCR and conventional PCR assays. As summarized in Table 4, for 75 cloacal swabs evaluated samples, 10 (13.33%) were MDPV-positive by qPCR assay. However, cPCR results showed 7 were MDPV-positive. For embryonic samples, 4 of 20 (20.0%) were MDPV-positive by qPCR assay and 2 of 20 (10.0%) were MDPV-positive by cPCR assay. For newly hatched ducklings, 5 of 20 (25.0%) were MDPV-positive by qPCR assay and 4 of 20 (20.0%) were MDPV-positive by cPCR assay, respectively. The samples tested with MDPV-positive by cPCR assay, were also tested MDPV-positive by qPCR assay. The viral DNA copy numbers in the positive samples when detecting MDPV DNAs were listed in Table 4. These findings provide evidence of possible vertical transmission of MDPV.

**Table 4:** Clinical samples tested for MDPV infection by conventional PCR and TaqMan-based PCR for MDPV detection

Samples	Region	Number of samples	Number of MDPV positive samples		Copy number for positive (copies/μL)	
			cPCR	qPCR	Both	Only
Cloacal Swabs	Fujian	15	2	4	7.45 × 10^4^, 1.72× 10^5^	3.59 × 10^1^, 8.24 × 10^1^
	Jiangxi	15	1	1	4.45 × 10^4^	–
	Guangdong	15	2	3	9.72× 10^3^, 2.45 × 10^4^	1.74 × 10^2^
	Jiangsu	15	1	1	8.61 × 10^4^	–
	Zhejiang	15	1	1	1.07× 10^4^	–
Embryos	Fujian	20	2	4	2.89 × 10^3^, 5.72× 10^3^	7.94× 10^1^, 1.18 × 10^2^
Newly hatched Muscovy ducklings	Fujian	20	4	5	3.89 × 10^3^, 6.02× 10^3^, 9.17× 10^3^, 2.21× 10^4^	9.47 × 10^1^

cPCR means conventional PCR; qPCR means TaqMan-based real-time PCR; Both means the samples were tested both cPCR-positive and qPCR-positive; Only means the samples were tested only by qPCR-positive. “-” means no only qPCR sample available

### Sequences analysis

A total of 13 (7 from cloacal swabs, 2 from embryonic samples, and 4 from newly hatched ducklings) amplicons from cPCR positive were harvested, purified, T-A cloned and then sequenced. The cloned sequences shared ≥99.1% nucleotide identity with MDPV (strain FM). Moreover, the cloned sequences from cPCR positive samples could specifically (100%) match the primers (MDPV-qF and MDPV-qR) and probe (MDPV-qP) listed in Table 2.

## Discussion

The current methods for the detection of MDPV, such as virus isolation, immunological-based assays, and electron microscopy, have proven to be laborious and time-consuming. Conventional PCR technology has been used for differentiation between MDPV and GPV, and the process includes restriction enzyme digestion, agarose gel electrophoresis, and DNA sequencing [12, 24]. Moreover, the PCR method for the specific detection of MDPV requires high precision primer design. Loop-mediated isothermal amplification (LAMP) [25] and an aptamer by label-free aptasensor [26] against MDPV for a highly sensitive, rapid visual detection was designed that targeted the VP3 gene of MDPVs. The VP3 protein is the most variable and abundant protein of MDPV. VP3 can induce neutralizing antibodies in GPV- or MDPV-infected waterfowl and confers protective cross-immunity in waterfowl parvoviruses [13, 27–30]. Moreover, VP3 has been associated with many genetic variations because of virus evolution selection pressure [6, 7, 31–33]. In addition, due to lack of information on the complete genomic sequence data of GPVs, especially MDPVs, false results can be obtained if primers are designed from unsuitable regions.

The real-time PCR technology has advantages with its remarkable sensitivity, specificity, reproducibility, visualization results, time-saving benefits, high-throughput analysis and less-contamination potential compared to other diagnostic methods, that have been widely used for viral pathogenesis research and epidemiology surveillance [16–21]. Woźniakowski et al. developed a TaqMan-based real-time PCR method for GPVs and MDPVs in which the designed primers and TaqMan probe for real-time PCR were complementary to GPV and MDPV inverted terminal repeats region (ITR) [34]. The calculation of results is often confounded due to the two ITR repeat regions at the 5′- and 3′-terminus in both the GPV and MDPV genomes. Moreover, mutation and deletion have been found in ITR of both GPVs and MDPVs, which may cause high risk of failure for the specific detection of MDPV infection [35–37]. In this study, we compared a total of 52 NS gene sequences (including 15 MDPVs and 37 GPVs) retrieved from the GenBank. We found that within the MDPV cluster, the samples shared higher nucleotide identity (more or equal than 98.0%) than within GPV cluster (more or equal than 93.3%). In addition, between GPV cluster and MDPV cluster, the NS gene homology ranged from 80.8 to 83.4%. These data indicate that false results may be obtained if these primers are designed at the NS gene-specific regions. These NS genetic comparison data allowed us to identify the MDPV-specific suitable regions, which can be used to establish a TaqMan-based real-time PCR assay for the detection of MDPV with more precision.

In this study, we clearly developed and evaluated the applicability of TaqMan-based real-time PCR for the detection and quantification of MDPV. The data of the qPCR assay was performed over a wide dynamic range with no fluorescence signal can be observed from other duck-derived pathogens, and low intra- and inter-assay variation (less than 2.21%). Field samples from cloacal swabs detection and those used in previous studies of MDPV infection provided the evidence that MDPV can be horizontally transmitted. Furthermore, the positive fluorescence signals observed in embryos and newly hatched Muscovy ducklings support the view that MDPV could be possible vertically transmitted as well as N-GPV [23].

## Conclusions

A specific and reliable TaqMan-based real-time PCR for the detection and quantification of MDPV was developed, because of significantly conserved region of NS genes between MDPVs and GPVs were selected for primers and probe design. The detection of the MDPV-positive in Muscovy duck embryos and newly hatched Muscovy ducklings, provide evidence of possible vertical transmission of MDPV from breeding Muscovy ducks to Muscovy ducklings.

## Abbreviations

AIV: avian influenza virus;
ATmV: avian Tembusu virus
BLAST: Basic Local Alignment Search Tool
cPCR: conventional PCR
Ct: threshold cycle
CV: Coefficient of variation
DAdV-A: Duck adenovirus A
DEV: Duck enteritis virus
DHAV: Duck hepatitis virus
GPV: Goose parvovirus
ICTV: International Committee on Taxonomy of Viruses
ITR: Inverted terminal repeats
LAMP: Loop-mediated isothermal amplification
MDPV: Muscovy duck parvovirus
MDRV: Muscovy duck reovirus
N-GPV: Novel goose parvovirus
NS: Nonstructural
ORF: Open reading frame
*P.M.*: Pasteurellamultocida
qPCR: real-time quantitative PCR
*R.A.*: Rimerella anatipstifer
R^2^: correlation
*S.S.*: Salmonella spp
